# Clinical and Patient‐Reported Outcomes in Grade III Furcations: A Randomized Feasibility Trial With SMART Design

**DOI:** 10.1111/jcpe.14134

**Published:** 2025-06-04

**Authors:** Priya Bahal, Pasquale Santamaria, Zainab Malaki, Jeremy Redman, Luigi Nibali

**Affiliations:** ^1^ Periodontology Unit, Centre for Host Microbiome Interactions, Dental Institute King's College London London UK; ^2^ Lewisham London UK

**Keywords:** furcation, periodontology, SMART

## Abstract

**Aim:**

To assess the feasibility of applying sequential, multiple assignment, randomized trial (SMART) design in periodontology and to assess the response of grade III furcation‐involved molars to various treatments.

**Methods:**

A member of the public served as a co‐investigator. Twenty participants with at least one grade III furcation‐involved molar tooth were randomized to either non‐surgical periodontal treatment (NSPT) or open‐flap debridement (OFD). They were reassessed 6 months later, where ‘successful response’ to therapy was defined with a ‘combined outcome’ incorporating patient‐reported and clinical parameters. Based on SMART principles, non‐responders underwent further treatment and were followed up to 12 months.

**Results:**

All study feasibility criteria were met, except ‘planned recruitment rate’, which was slower than expected. At the 6‐month review, those who underwent OFD showed a greater reduction in probing pocket depth (PPD) compared to those who received NSPT (*p* = < 0.01). ‘Success’ was reached by 6 out of 10 NSPT participants and by 7 out of 10 OFD participants. Non‐responders were further randomized and followed up to 12 months.

**Conclusion:**

This SMART design is feasible in periodontology trials. This study demonstrates its advantages and limitations along with clinical and patient‐reported data of grade III furcations treated surgically or non‐surgically.

## 1 Introduction

Furcation involvement (FI), classified by horizontal and vertical bone loss (Hamp, Nyman, and Lindhe 1975; Tarnow and Fletcher 1984), significantly increases the risk of tooth loss (Nibali et al. 2016). This risk further increases with greater horizontal (Nibali et al. 2016, 2017) and vertical bone loss (Nibali, Tomlins, and Akcalı 2018; Tonetti, Christiansen, and Cortellini 2017).

Once FI is established, treatment options include non‐surgical treatment, resective and regenerative approaches or extraction (Nibali et al. 2016). However, these teeth often do not respond well to non‐surgical therapy (Loos, Claffey, and Egelberg 1988). Advanced FI, especially grade III, is particularly challenging when attempting regenerative therapies (Pontoriero and Lindhe 1995). Despite being aware of these difficulties and the prevalence of FI, no clear treatment guidelines exist (Dommisch et al. 2020).

There is a need for evidence for advanced FI treatment and a greater shift towards personalized periodontology, considering individual patient factors for tailored treatment plans (Duarte and Spencer 2016). The sequential, multiple assignment, randomized trial (SMART) study design offers this with more flexibility and external validity compared to traditional randomized controlled trials (RCTs) (Almirall et al. 2014). At present, personalized medicine designs are uncommon in dental research, and as no RCTs on grade III FI exist, initial feasibility data on recruitment, willingness to participate and treatment response rates are needed.

Therefore, the aim of this feasibility study was to assess the feasibility of applying the SMART design in periodontology and to assess the response of grade III furcation‐involved molars to various treatments by using a composite outcome including clinical and patient‐reported data.

## 2 Materials and Methods

This was a single‐centre, single‐masked, randomized controlled feasibility trial with SMART design with 12‐month follow‐up to assess the response to treatment for grade III periodontal furcation‐involved molars (*n* = 20). A patient champion (author J.R.) was involved in the study as co‐investigator, giving advice on study design, study conduct, data interpretation and dissemination, particularly contributing to aspects related to patient‐reported outcomes. The CONSORT checklist is reported as Supporting Information 1.

### 2.1 Experimental Design

Ethical approval was obtained by the Health Research Authority (HRA) and Health and Care Research Wales (HCRW) Ethics Committee (reference 21/EE/0256), and the study was conducted according to the principles outlined in the Declaration of Helsinki on experimentation involving human subjects. The study was registered on clinicaltrials.gov on 24 January 2022 (reference ID 23012022; https://clinicaltrials.gov/study/NCT05237739?cond=treatment%20of%20advanced%20grade%20III%20periodontal%20furcation%20lesions&rank=1; identification number: NCT05237739). Each patient gave written consent to take part in the study.

### 2.2 Population Screening

Potential study subjects were identified from those referred to the periodontal department at Guy's Dental Hospital. Each underwent a complete periodontal examination, including medical and dental history, intra‐oral examination, full‐mouth periodontal probing and periapical radiographs. After making a periodontal diagnosis, subjects who met the inclusion criteria received a written information sheet explaining the protocol and were invited to participate.

The study included participants (i) aged 18–70, (ii) with a diagnosis of periodontitis stage III or IV, grade A, B or C (Tonetti, Greenwell, and Kornman 2018), (iii) with the presence of ≥ 1 tooth with FI grade III (Hamp, Nyman, and Lindhe 1975) grade C (Tarnow and Fletcher 1984) without any restorative problems and mobility less than grade III, (iv) with no ongoing endodontic pathology (as examined by the study clinician), (v) able to give consent to study participation and (vi) in whom non‐surgical periodontal therapy was performed to the study site within the last 6 months. Exclusion criteria were (i) smoking (current or in the past 5 years), (ii) a medical history including diabetes or other serious medical conditions or transmittable diseases, (iii) requiring prophylactic antibiotic coverage prior to invasive dental procedures, (iv) anti‐inflammatory or anticoagulant therapy within 1 month of the baseline exam, (v) systemic antibiotic therapy within 3 months of the baseline exam, (vi) a history of alcohol or drug abuse, (vii) self‐reported pregnancy or lactation, (viii) other severe acute or chronic medical or psychiatric condition or laboratory abnormality that, according to the investigator, might increase the risk associated with trial participation and (x) previous surgical periodontal therapy performed to the study site. In case of multiple suitable molars per patient, the one with worst pocket depth was selected as the study site.

### 2.3 Pre‐Treatment

All participants had undergone a course of professional mechanical plaque debridement prior to entering the study within the previous 6 months. Following consent, at baseline, self‐reported patient medical and smoking histories were checked. Clinical parameters were assessed by gentle probing using a UNC‐15 periodontal probe and a Nabers probe for FI. A furcation ‘test site’ was defined as a tooth with III C FI, which had no restorative problems, endodontic pathology or mobility score III.

### 2.4 Sample Size Calculation

Due to the lack of previous SMART trials in periodontology, no power calculation was possible. Thus, a convenience sample of 20 participants was recruited. The study investigated recruitment rates, willingness to participate and be randomized and the frequency of ‘responders’ to different treatments.

### 2.5 Randomization and Allocation Concealment

Participants were assigned a subject number, and data were collected at the baseline visit. They were then randomly assigned to receive either non‐surgical periodontal treatment (NSPT) or open‐flap debridement (OFD) using simple randomization based on a computer‐generated randomization table (www.sealedenvelope.com) prepared by an independent staff member, to ensure equal distribution between treatments. There was no stratification for patient characteristics.

Treatment allocations were kept in numbered envelopes accessible only to the study clinicians. When a participant was ready for treatment, the clinician opened the appropriate envelope and arranged the treatment. The randomization code was concealed from the study examiner until after data analysis.

**FIGURE 1:**
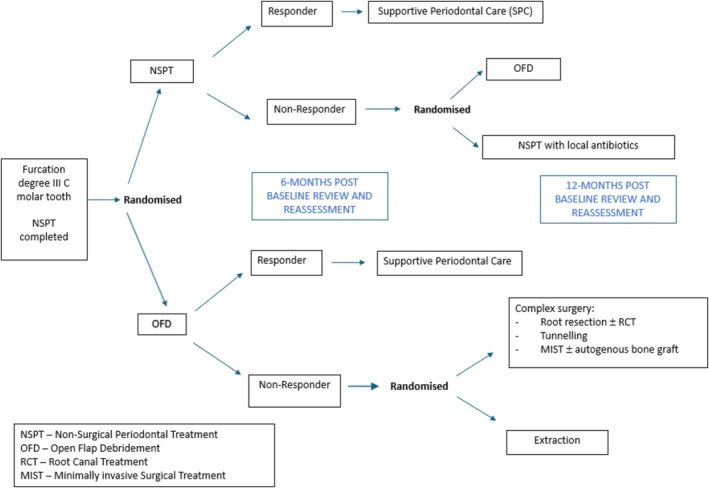
Flow diagram showing the different phases of the study design.

After a reassessment at 6 months, clinical and patient‐reported outcome measures (PROMS) were collected. Participants deemed ‘non‐responders’ (as they did not meet the ‘success’ criteria) were randomized again according to their initial allocation. Each participant had a predetermined randomization pathway due to the staggered 6‐month assessments. Study visits and timelines are summarized in Figure 1.

Clinical details about the NSPT and OFD which were carried at treatment 1 are provided in Supporting Information 2a,b.

### 2.6 ‘Success’ Criteria

‘Success’ was defined based on a combination of clinical and participant‐reported parameters, as explained below:

- Clinical parameter for ‘success’: absence of PPD ≥ 6 mm with bleeding on probing (BOP) (Loos and Needleman 2020)
- Participant‐reported parameter for ‘success’: ≤ 1 ‘yes’ response to furcation specific questionnaire.

Both parameters needed to be fulfilled for the definition of ‘success’ to be met. Examples of possible responses and outcomes are provided in Supporting Information 3.

### 2.7 Clinical Parameters

The full‐mouth clinical parameters recorded were plaque, probing pocket depths (PPD) and recession (REC). Further details are provided in Supporting Information 4.

### 2.8 Calibration

One of the authors (P.S.) completed measurements at the baseline, 6‐ and 12‐month visits on all participants. Intra‐examiner calibration was carried out prior to the first study visit. Further details are provided in Supporting Information 5.

### 2.9 Patient‐Reported Outcome Measures

Participants independently completed the three questions listed below at the baseline, 6‐ and 12‐month appointments. The following questions were in relation to their test tooth:

1. Do you have any discomfort from the tooth with furcation involvement? (yes/no).
2. Does the gum bleed around the tooth with furcation involvement? (yes/no).
3. Is the tooth sensitive to cold or warm temperatures? (yes/no).

### 2.10 Re‐Assessment Examinations

Early healing was evaluated at 1 and 3 months after treatment 1 and 2 by Z.M. or P.B. Only the study examiner (P.S.) conducted the reassessments at 6 and 12 months after treatment 1.

### 2.11 Feasibility Criteria

Specific ‘progression’ criteria were set to determine if a definitive trial could be considered ‘feasible’, including recruitment of at least four patients per month, > 80% of patients retained in the study, > 80% of patients willing to be randomized and ability to combine clinical and patient‐reported outcomes (both not being identical in ‘success’, as otherwise one of the outcomes would be redundant).

### 2.12 Statistical Analysis

Data from all included patients were entered into a spreadsheet and proofread for entry errors. Data analysis was performed using R Studio Core Team 2020. A descriptive analysis was carried out to assess how many cases reached clinical and patient‐reported ‘success’ (main outcome) at 6 and 12 months. Exploratory analyses of achievement of success and clinical periodontal data were also assessed for patients divided by study group (test vs. control).

## 3 Results

### 3.1 Patient Flow

**FIGURE 2:**
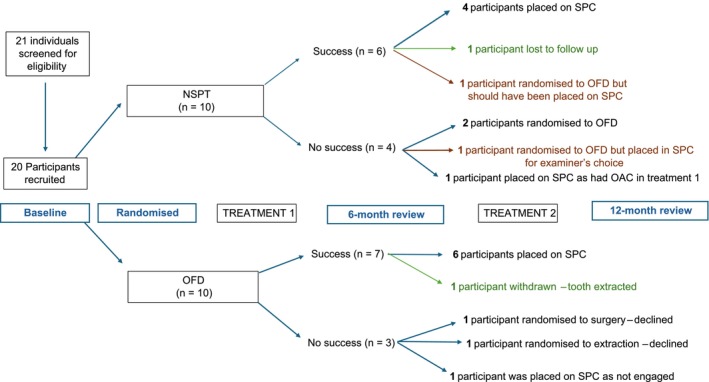
Flow diagram summarizing participant flow through the study.

Participants were recruited from June 2022 to March 2023. Study visits took place from June 2022 to March 2024. Twenty‐one individuals were screened for eligibility, of whom 20 were willing to participate and were recruited into the study. One participant was unable to proceed further in the trial as she became pregnant. Figure 2 shows the study diagram from baseline to 12 months. As per protocol, 10 participants were randomized to NSPT while the other 10 underwent OFD on the test teeth.

At the 6‐month review of the 10 participants who underwent NSPT, 6 achieved ‘success’ while the other 4 did not. For those who underwent OFD, at the 6‐month review, 7 reached ‘success’ while 3 did not.

As per SMART protocol, the responders to NSPT and OFD then entered supportive periodontal care (SPC), which was carried out at 1 and 3 months after the 6‐month review. The non‐responders, however, were further randomized to a more invasive treatment (based on the principle of ‘augment’, where an additional, more advanced procedure is offered).

Of the four non‐responders to NSPT, two were randomized to OFD, and this was completed uneventfully. A third non‐responder would have been randomized to further treatment; however, the participant suffered an oro‐antral communication (OAC) during treatment 1 and so it was decided that it was in the patient's best interests to enter SPC for the remainder of the trial. The final non‐responder should have been randomized to further treatment; however, as the ‘lack of success’ was attributed to traumatic interdental brushing in the area and no PPD > 4 mm was detected, the study examiner decided to enter the patient in SPC.

Of the three non‐responders to OFD, one was randomized to extraction of the test tooth, which was declined by the patient. The two remaining non‐responders were randomized to further surgery. However, one did not wish to undergo another surgical procedure; and for the other, it was deemed not in the best interest of the patient to perform another surgery owing to the sub‐optimal level of plaque control. This resulted in none of the participants who received OFD at treatment 1 undergoing a second treatment, despite not having achieved the ‘success’ criteria as per the study protocol. These three teeth were consequently entered into SPC for the duration of the study. Of note, one responder to NSPT should have entered SPC but was mistakenly labelled as a non‐responder by an examiner and so was randomized to and received OFD.

### 3.2 Baseline Characteristics

Table 1 shows the baseline demographic and clinical characteristics of all participants. All participants were diagnosed with either stage III C or IV C periodontitis, with an equal distribution of generalized and localized cases. All 20 participants returned for their 6‐month visit, while 18 returned for the 12‐month review. Reasons for non‐attendance at 12 months were either the test tooth being extracted due to unrestorable caries (*n* = 1) or the participant being lost to follow‐up (*n* = 1).

**TABLE 1:** Baseline demographic and clinical characteristics of all participants.

Age	56 ± 10.1	
BMI	27.7 ± 4.4	
Gender	Male	9 (45%)
	Female	11 (55%)
Ethnicity	Caucasian	8 (40%)
	Asian	6 (30%)
	Afro‐Caribbean	6 (30%)
Periodontal diagnosis stage	III	15
	IV	5
Periodontal diagnosis grade	C	20
Periodontal diagnosis extent	Localized	10
	Generalized	10
FMPS (%)		14.3 ± 7.8
FMBS (%)		21.6 ± 12.4

*Note:* Averages are shown with standard deviation (SD).

### 3.3 Feasibility/Progression Criteria

The percentage of participants who showed coincident and non‐coincident clinical and patient‐reported outcomes at the 6‐ and 12‐month reassessment visits is shown in Supporting Information 6.

Recruitment rate was slower than expected (1.3/month), while the targets of > 80% retention in the study and > 80% willingness to be randomized targets were achieved, as only one patient was lost to follow‐up and two refused the random allocation at the 6‐month timepoint. There was no perfect agreement between ‘clinical success’ and ‘patient‐reported success’ (coinciding only in 12 out of 20 cases at 6 months and 14 out of 18 cases at 12 months), showing the value in adding the patient‐reported element.

Table 2 shows the mean average of PPD, REC and clinical attachment level (CAL) with differences between timepoints. A gradual decrease in full‐mouth PPD and CAL was detected throughout the study in all teeth, while a considerable decrease in PPD and CAL and an increase in REC were detected in the test teeth. Overall, 80% and 83% of test teeth achieved clinical ‘success’ at 6 and 12 months, respectively, while the patient‐reported ‘success’ was 80% at both 6 and 12 months.

**TABLE 2:** Average of probing pocket depths (PPD), clinical attachment level (CAL), recession (REC) and probing depths with differences between time points.

Clinical measurements	Baseline	6 months	12 months	Δ 0–6 months	Δ 0–12 months
				*p*	*p*
Average PPD (mm)	2.9 ± 1.6	2.6 ± 1.4	2.4 ± 1.3	< 0.001	< 0.001
Average REC (mm)	1.3 ± 1.6	1.4 ± 1.7	1.2 ± 1.5	0.142	< 0.001
Average CAL (mm)	4.2 ± 2.7	4.0 ± 2.6	3.5 ± 2.4	< 0.001	< 0.001
Number of PPDs ≥ 5 mm (average per participant)	20.4 ± 14	14.7 ± 12.7	11.8 ± 7.8	0.186	0.046
Average of deepest PPD at test teeth with corresponding REC and CAL					
PPD (mm)	7.1 ± 2.0	5.2 ± 1.8	4.7 ± 1.5	< 0.001	< 0.001
REC (mm)	2.4 ± 2.3	3.1 ± 2.2	3.3 ± 2.6	0.175	0.020
CAL (mm)	9.5 ± 2.5	8.4 ± 2.5	7.9 ± 3.1	0.06	0.100

Table 3 reports data relative to the test teeth at baseline and 6 months according to treatment allocation (NSPT vs. OFD). There was a greater reduction in the deepest PPD at the test sites when OFD was completed compared to NSPT, with a reduction of 2.3 versus 1.4 mm, respectively. PPD reduction compared to baseline was only statistically significant for the OFD group (*p* < 0.01). This difference appeared to be maintained until the 12‐month review, despite only the non‐responders who underwent NSPT at treatment 1 undergoing further treatment. These results show that the initial reduction in PPD in grade III molar teeth appears to be more favourable with a surgical than a non‐surgical approach despite a ‘SMART’ approach at 6 months.

**TABLE 3:** Average of deepest pocket probing at test sites for those who received NSPT or OFD at treatment 1.

Clinical measurements	Treatment 1	Baseline	6 months	12 months	Δ 0–6 months	*p*	Δ 6–12 months	Δ 0–12 months
PPD deepest site for test tooth (mm)	NSPT	6.8 ± 1.48	5.4 ± 1.96	5.1 ± 1.83	−1.4 ± 2.07	0.06	−0.3	−1.7
	OFD	7.3 ± 2.54	5.0 ± 1.63	4.2 ± 0.97	−2.3 ± 2.06	< 0.01	−0.8	−3.1

**FIGURE 3:**
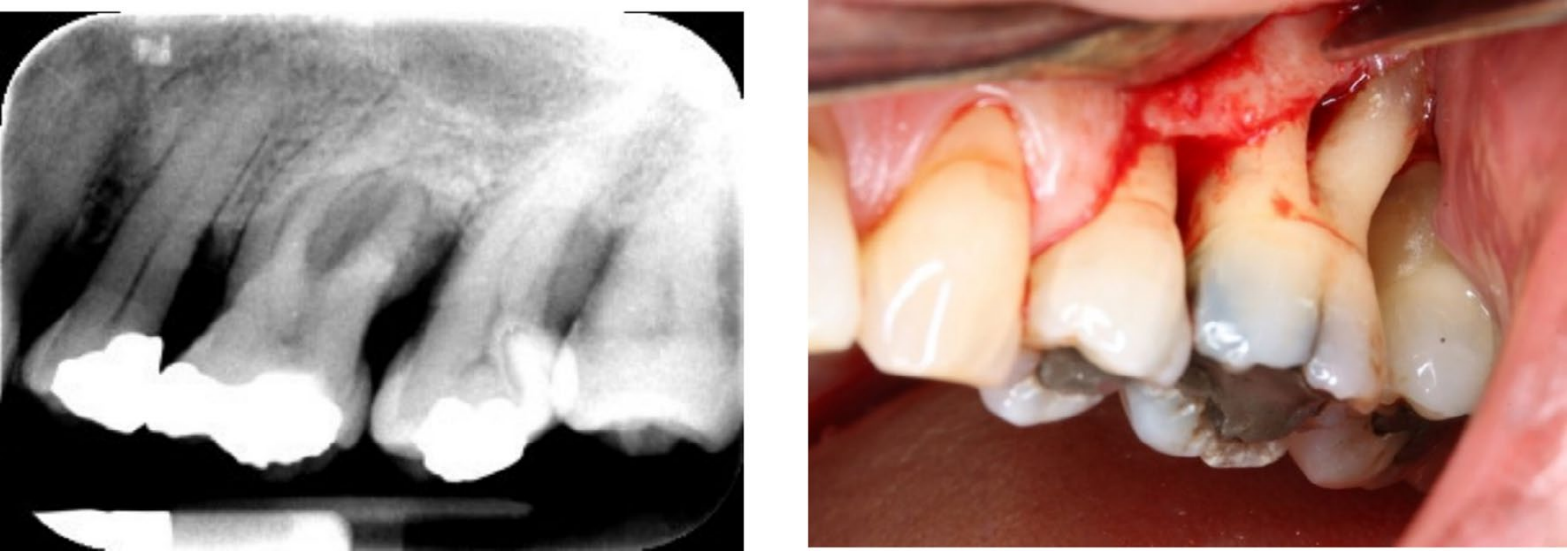
(a) Pre‐operative periapical radiograph of UL6 at baseline. (b) Intra‐operative photo of UL6 which was randomized to NSPT for treatment 1 and following a lack of success at the 6‐month review, was randomized to OFD for treatment 2.

An example of a case that was randomized to NSPT for treatment 1 was deemed unsuccessful at 6 months and then randomized to OFD for treatment 2 (Figure 3a,b).

## 4 Discussion

This feasibility study showed that the SMART design is able to meet the criteria for participant retention and willingness to be randomized, demonstrating its potential for future periodontology trials. This study also showed how clinical and patient‐reported outcomes can be combined to define ‘success’ and provides initial comparative data for non‐surgical and surgical treatments of grade III furcation involvement.

As health care shifts towards personalized care, the SMART design offers several research advantages. Periodontal treatment typically starts with less invasive methods, followed by healing and reassessment before further treatment. The European Federation of Periodontology's (EFP) stepwise approach suggests starting with less invasive NSPT, followed by reassessment and potentially more complex treatments, like the SMART design. It is therefore logical for periodontal research to align with the stepwise clinical approach. SMART studies, with their reassessment and re‐randomization features, allow for the study of treatment sequences (Moodie, Karran, and Shortreed 2016), which is particularly useful for grade III FI treatment where no RCTs exist and treatment consensus is lacking.

Although the main outcomes of this feasibility study were achieved, there was a limitation with the evaluation of the success criteria at 6 months. In one case, the reason for the success criteria not being met at 6 months was attributed to traumatic interdental brushing in the area. In this case, as no PPD > 4 mm was detected, the study examiner decided to enter the patient into SPC. Furthermore, an examiner's mistake occurred whereby a participant had no concerns with the test tooth, and the deepest probing depth of 6 mm with no bleeding was present. According to the protocol, this would be a responding participant who would enter SPC; however, it was marked as a non‐responder, and the participant underwent OFD, demonstrating the complexity and potential for error in SMART studies. Interestingly, based on the EFP guidelines, had the patient presented in the same way in the clinical scenario, a treatment such as OFD would have been offered.

The recruitment rate of > 4 participants/month was not achieved; however, this is not a limitation specific to the SMART design. It was likely to be associated with the study's inclusion criteria and the clinical challenges associated with confirming a grade III furcation‐involved molar, particularly immediately after the pandemic, when clinic numbers had not gone back to full capacity.

At 6 months and in line with the literature (Sanz‐Sánchez et al. 2020), there was a greater reduction in PPD in participants who underwent OFD compared to those who underwent NSPT with reductions of 2.3 and 1.4 mm, respectively. Interestingly, these differences between OFD and NSPT were still present at 12 months despite the re‐modulation of treatment at 6 months. As mentioned above, as no RCT specific to the treatment of grade III FI molar teeth exists, this study could serve to provide data for sample size calculations for future RCTs on the topic, regardless of whether the SMART design is used.

Although participants were willing to be randomized, uptake for a second surgical procedure was low. The two participants eligible for further treatment after OFD (either periodontal surgery or extraction) declined. This highlights a limitation of the SMART design, as without participant consent, treatments cannot proceed, leading to potential dropouts, especially after an initial surgical treatment. The equal weighting given to the clinical and PROMS results presented decision‐oriented challenges. For instance, a participant might have positive PROMs but not met clinical success criteria, classifying him or her as a non‐responder and suggesting further treatment. Conversely, a tooth procedure might be clinically successful, but the patient might still desire additional treatment. In this research scenario, giving both domains equal importance can result in challenges that are otherwise not encountered in the clinical scenario where a more dynamic relationship exists. This may suggest that the 50/50 approach is not suitable for a composite outcome measure, and it may be necessary to give greater weighting to the clinical or PROMS result. Furthermore, there would be merit in simplifying the composite measures for success, as the study examiner had difficulty in objectively adhering to the composite criteria but instead bought a subjective opinion when deciding if a participant was deemed a responder or a non‐responder.

A novel aspect was the involvement of a ‘patient champion’ (co‐investigator/author J.R.), who was a member of the public interested in supporting the study. The patient champion proofread study documents, including participant information sheets, informed consent forms and questionnaires and was also able to provide insights into how participants might perceive the study and randomization processes. The inclusion of patient champions or a ‘patient with lived experience’ (PWLE) in research has only recently been recognized in the literature (Needleman et al. 2023). While traditional periodontal parameters like PPD and BOP provide prognostic information, incorporating PROMs and involving a patient champion or expert patient offers a more comprehensive approach, with the potential to increase external validity.

Study limitations include the small sample size (pilot), being a single‐site study and the previously mentioned examiner‐ or patient‐driven deviations from re‐treatment allocation.

## 5 Conclusion

To summarize, this was the first RCT in periodontology, to our knowledge, to adopt a SMART design and show its feasibility, show the importance of combining clinical and patient‐reported data to define ‘success’, report clinical data on surgical versus non‐surgical approaches for the treatment of grade III furcation‐involved molars and, finally, include a patient champion as a co‐investigator.

Taken together, the SMART study design offers many advantages, yet its use in dentistry remains limited. This may be due to the need for extensive planning, challenges in securing funding, interpreting results or lack of awareness about the design (Alshamsi, Mehta, and Nibali 2021). We hope this study will inspire more research with greater external validity and a personalized approach in periodontology.

## Author Contributions

**Priya Bahal:** conceptualization, investigation, data analysis, writing – original draft. **Pasquale Santamaria:** investigation, writing – review and editing. **Zainab Malaki:** investigation, writing – review and editing. **Jeremy Redman:** conceptualization, writing – review and editing. **Luigi Nibali:** conceptualization, methodology, data analysis, writing – review and editing, supervision.

## Conflicts of Interest

The authors declare no conflicts of interest.

## Supporting information


**Supporting Information 1.** CONSORT checklist.
**Supporting Information 2**. (a) Clinical details about NSPT (non‐surgical periodontal treatment). (b) Clinical details about OFD (open‐flap debridement).
**Supporting Information 3**. Outcomes of clinical and patient‐reported responses at 6‐months.
**Supporting Information 4**. Recording of clinical parameters.
**Supporting Information 5**. Intra‐examiner calibration.
**Supporting Information 6**. Graph showing the percentage of participants who demonstrated coincident and non‐coincident clinical and patient‐reported outcomes at the 6‐ and 12‐month reassessment visits.

## Data Availability

The data that support the findings of this study are available from the corresponding author upon reasonable request.
