# Supplementary material for: Clinical and Patient‐Reported Outcomes in Grade III Furcations: A Randomized Feasibility Trial With SMART Design
Source: J Clin Periodontol. 2025 Jun 4;52(7):932–9. doi: 10.1111/jcpe.14134 (PMC12176455; doi:10.1111/jcpe.14134)
Supplement: Supplementary file 1 — Supporting Information 1. CONSORT checklist. Supporting Information 2. (a) Clinical details about NSPT (non‐surgical periodontal treatment). (b) Clinical details about OFD (open‐flap debridement). Supporting Information 3. Outcomes of clinical and patient‐reported responses at 6‐months. Supporting Information 4. Recording of clinical parameters. Supporting Information 5. Intra‐examiner calibration. Supporting Information 6. Graph showing the percentage of participants who demonstrated coincident and non‐coincident clinical and patient‐reported outcomes at the 6‐ and 12‐month reassessment visits. [file JCPE-52-932-s001.docx]

1. **CONSORT Checklist**

**2a. Non-surgical periodontal therapy (NSPT)**

NSPT was initiated at least two weeks following the baseline screening examination for participants allocated to it. A standard cycle of periodontal therapy was completed on the test tooth under local anaesthesia (2% lidocaine hydrochloride 1:80, 000). Oral hygiene advice was given, along with supra- and sub-gingival mechanical instrumentation of the root surface. This treatment was carried out by one of two study clinicians (ZM and PB), separate from the study examiner (PS) using Gracey curettes and ultrasonic scalers. Treatment time was recorded for the visit

**2b. Open flap debridement (OFD)**

Participants allocated to OFD received treatment by one of the two study clinicians (ZM or PB) under local anaesthetic (2% lidocaine hydrochloride with adrenaline 1:80, 000) and intrasulcular incisions were made on the buccal and lingual/palatal aspects to reflect full-thickness flaps. The granulation tissue around the tooth was removed with the non-cutting edge of a suitable scaling instrument such as a curette, taking care not to damage the root surfaces. Thorough debridement of the furcation area was carried out by using piezoelectric / ultrasonic devices with thin tips and Gracey curettes as necessary. The surgical site was closed with 4/0 Vicryl Rapide (resorbable) simple interrupted sutures. Participants were given detailed post-operative instructions regarding care of the site. Treatment time was recorded for the visit.

1. **Outcomes of clinical and participant-reported responses at 6-months**

|  | Clinical response (absence of PPD ≥ 6mm with BOP) | Participant-reported response (≤ 1 ‘yes’ response to specific molar questionnaire) | Overall ‘responsive to treatment’ |
| --- | --- | --- | --- |
| **CASE 1** | Yes | Yes | Yes |
| **CASE 2** | Yes | No | No |
| **CASE 3** | No | Yes | No |

Table demonstrating possible combinations of clinical response and participant questionnaire responses at the 6-month assessment appointment with the final column indicating the overall response to treatment result to which they would then be allocated.

1. **Recording of clinical parameters**The calibrated examiner assessed the following periodontal measurements at six tooth sites: dichotomous full mouth plaque scores (FMPS) (Guerrero et al., 2005), full mouth probing pocket depth (PPD), recession (REC) of the gingival margin from the cemento-enamel junction (CEJ) with measurements rounded to the nearest millimetre. REC was recorded as a positive number if the free gingival margin was apical to the CEJ, while it was a negative value if it was coronal to the CEJ. Bleeding on probing Full Mouth Bleeding Score (FMBS) (Ainamo & Bay 1975), tooth mobility (Laster et al. 1975) and FI (Hamp et al., 1975) were also assessed. Full Mouth Plaque Score (FMPS) was recorded by giving the participant a plaque disclosing tablet to chew on and then assigning a binary score to each surface (1 for plaque present and 0 for plaque absent). The percentage of total tooth surfaces on which plaque was present was recorded. Clinical attachment level (CAL) was calculated as PPD + REC.
2. **Intra-examiner calibration**

Following initial training, the examiner performed repeated examinations on 10 subjects for PPD, REC and FI with at least 15 minutes’ separation. Upon completion of all measurements, intra-examiner repeatability for PPD measurements was assessed. The calibration for continuous variables (PPD and REC) was analysed with the Bland–Altman graph and by calculation of Kappa agreement coefficient. The resulting coefficient of agreement was significant (0.8 SE 0.02; p = .001), considering that a coefficient of repeatability less than ±2 mm in 95% of the cases was considered acceptable. For FI, an Intraclass correlation analysis (ICC) was performed using the Cohen’s Kappa for qualitative variables, obtaining an ICC > 0.78.

1. **Graph showing the percentage of participants who demonstrated coincident and non-coincident clinical and patient-reported outcomes at the 6- and 12-month reassessment visits.**

REFERENCES

Ainamo J, Bay I. Problems and proposals for recording gingivitis and plaque. Int Dent J. 1975;25(4):229-235

Guerrero, A., Griffiths, G. S., Nibali, L., Suvan, J., Moles, D. R., Laurell, L., & Tonetti, M. S. (2005). Adjunctive benefits of systemic amoxicillin and metronidazole in non-surgical treatment of generalized aggressive periodontitis: a randomized placebo-controlled clinical trial. Journal of Clinical Periodontology, 32(10), 1096–1107.

Hamp, S.-E., Nyman, S., & Lindhe, J. (1975). Periodontal Treatment of multirooted teeth. Results after 5 years. (pp. 126–135). Journal of Clinical Periodontology.

Laster L, Laudenbach KW, Stoller NH. An evaluation of clinical tooth mobility measurements. J Periodontol. 1975;46(10):603-607. doi:10.1902/jop.1975.46.10.603
